# Report on the National Eye Institute Audacious Goals Initiative: Replacement of Retinal Ganglion Cells from Endogenous Cell Sources

**DOI:** 10.1167/tvst.6.2.5

**Published:** 2017-03-15

**Authors:** Monica L. Vetter, Peter F. Hitchcock, and the AGI workshop participants

**Affiliations:** 1 Department of Neurobiology and Anatomy, University of Utah, Salt Lake City, UT, USA; 2 Department of Ophthalmology and Visual Sciences, University of Michigan, Ann Arbor, MI, USA

**Keywords:** retinal ganglion cell biology, Muller glia, cell-based therapies, endogenous replacement strategies

## Abstract

This report emerges from a workshop convened by the National Eye Institute (NEI) as part of the “Audacious Goals Initiative” (AGI). The workshop addressed the replacement of retinal ganglion cells (RGCs) from exogenous and endogenous sources, and sought to identify the gaps in our knowledge and barriers to progress in devising cellular replacement therapies for diseases where RGCs die. Here, we briefly review relevant literature regarding common diseases associated with RGC death, the genesis of RGCs in vivo, strategies for generating transplantable RGCs in vitro, and potential endogenous cellular sources to regenerate these cells. These topics provided the clinical and scientific context for the discussion among the workshop participants and are relevant to efforts that may lead to therapeutic approaches for replacing RGCs. This report also summarizes the content of the workshop discussion, which focused on: (1) cell sources for RGC replacement and regeneration, (2) optimizing integration, survival, and synaptogenesis of new RGCs, and (3) approaches for assessing the outcomes of RGC replacement therapies. We conclude this report with a summary of recommendations, based on the workshop discussions, which may guide vision scientists seeking to develop therapies for replacing RGCs in humans.

## Introduction

In developed countries, losing vision is among the most feared ailments, and across all countries blindness creates enormous economic and social burdens^1^ (and references cited therein). In developing countries, the majority of visual disability is due to uncorrected refractory errors and cataracts.^2–4^ However, in developed countries, where the barriers to accessing modern medicine are low, visual disability results more commonly from diseases that result in the death of retinal neurons, principally photoreceptors and retinal ganglion cells. In humans, the death of these cells results in irreversible blindness. This bleak outcome is the motivation for developing multipronged approaches for treating blinding diseases.^5^ One approach is to transplant cells that are capable of replacing the depleted neurons. Another is to induce cells endogenous to the retina to regenerate supplementary neurons, a capacity already possessed by some cold-blooded vertebrates. Implicit in either approach are the requirements that new neurons are correctly specified, survive, and make functional synaptic connections that restore useful vision. The NEI, as part of the AGI, has convened a series of workshops focused on regenerating neurons and their synaptic connections within the human retina and visual system. The first three workshops addressed, respectively, regenerating the opic nerve,^6^ regenerating and integrating photoreceptors,^7^ and reconnecting neurons in the visual system.^8^ This is a report from the fourth workshop addressing the replacement of retinal ganglion cells (RGCs) from exogenous and endogenous sources.

### Diseases Associated with Loss of Retinal Ganglion Cells

Retinal ganglion cells integrate synaptic signals in the retina and transmit the resulting visual information to the brain. This visual information is carried by long axons that course over the surface of the retina, exit the eye through the optic disc (optic nerve head), and form the optic nerve. During their intraretinal course, the axons of RGCs are unmyelinated. Once axons exit the globe, they become myelinated, and the optic nerve then closely resembles other white matter tracts of the central nervous system.

Diseases that result in the death of RGCs are broadly categorized as optic neuropathies.^9,10^ The most common optic neuropathy is glaucoma, the leading cause of irreversible blindness worldwide.^11^ A recent population-based survey estimated that in the United States, glaucoma affects 2.3 million individuals 60 years of age and older.^12^ Glaucoma results in a progressive and selective loss of RGCs axons and death of the cell bodies.^13,14^ Interestingly, there can be a prolonged asymptomatic phase of glaucoma, and substantial ganglion cell death can occur in the absence of clinically detectable visual field defects.^15^ Treatment for glaucoma can decelerate but not overcome the progressive death of RGCs. Consistent with the progressive nature of this disease, the end stage of damage can result in loss of all RGCs.

Ischemic optic neuropathy is similar to a stroke within the optic nerve which injures axons, leading to the death of RGCs.^16–18^ As seen in strokes of other axonal tracts in the central nervous system, and unlike glaucoma, ischemic optic neuropathy has a rapid onset and variable degrees of injury, which can range from normal acuity with some visual field defects to profound defects in both acuity and visual fields. There are different forms of ischemic optic neuropathy, based on the pathogenic mechanism. Ischemic optic neuropathy due to underlying giant cell arteritis generally leads to more severe vision loss, compared with nonarteritic anterior ischemic optic neuropathy, and after occurring in one eye, can become bilateral within days.

The hereditary optic neuropathies are genetic disorders that result in simultaneous or sequential bilateral vision loss due to the death of RGCs.^19–22^ This disease may appear in children or adults and may be isolated to the optic nerve, such as in Leber hereditary optic neuropathy and dominant optic atrophy, or may be present in the optic nerve as a component of a more broadly distributed neurodegenerative process.^19^ In contrast to glaucoma, inherited optic neuropathies first present as central scotomas, indicating preferential death of RGCs that transmit high acuity, macular, and foveal vision.

Toxins, nutritional deficiency, or trauma can also create optic neuropathies that lead to the death of RGCs.^23–25^ Toxic optic neuropathies can result from drugs such as ethambutol or linezolid, methanol, or certain heavy metals. Vitamin B_12_ deficiency can cause an optic neuropathy in vegans or those who do not absorb dietary B_12_. Finally, it is well established that trauma to the mammalian optic nerve, such as from direct or indirect trauma, results in rapid death of RGCs.^26^

### Genesis of Retinal Ganglion Cells In Vivo

The targeted replacement of RGCs by transplanting cells or regenerating cells from endogenous sources will necessarily rely upon a fundamental understanding of the mechanisms that control the embryonic genesis of RGCs. Retinal histogenesis from undifferentiated progenitors relies on the coordinated interplay of intrinsic and extrinsic cellular signaling, which establishes patterns of cell proliferation, initiates the onset of cell cycle withdrawal, specifies neuronal identities, and governs cellular differentiation.^27,28^ The retina has long served as a model for delineating intrinsic cellular mechanisms, whereby multipotent progenitors produce discrete cell types. This work, in part, has led to the concept of hierarchical gene-regulatory networks. In its simplest form, a gene-regulatory network in the retina is the combination of transcription factors, and the target genes they regulate, which sequentially determine cell fates, neuronal differentiation, and the assembly of synaptic circuits.^29^ The most well-studied gene regulatory networks in the retina are those that produce rod and cone photoreceptors^30^ and RGCs.^31–33^

Initially, all retinal progenitors require the homeodomain transcription factor, Pax6, both to adopt retinal identities and to sustain proliferation. The first evidence of RGC specification is the expression of the bHLH transcription factor, Atoh7 (originally named Ath5), in progenitors as they exit the cell cycle.^34^ Atoh7 functions as a RGC competence factor, and the loss of Atoh7 results in retinas with largely normal architecture, but the nearly complete absence of ganglion cells.^35–37^ Subsequent to identifying Atoh7 in the gene-regulatory network for RGCs, efforts were begun to identify both its upstream regulators and downstream targets.^32,38^ A current model of this network places Atoh7 downstream of Pax6 and Hes1^32^ and upstream of the transcription factors, Pou4f2 and Isl1, which together are sufficient to specify RGC fates, but also function to regulate RGC differentiation.^39^ This gene regulatory network is sufficient to broadly specify RGCs, but does not account for the genesis of the known ganglion cell subtypes. There may be as many as 30 subtypes of RGCs in mouse, each with distinct morphology, synaptic inputs, functions, and central targets.^40,41^ A significant challenge that remains is to define RGC subtypes in humans, identify the gene networks that determine each, and establish their unique subtype-specific functional properties.

### Genesis of Retinal Ganglion Cells In Vitro

Knowledge gained from decades investigating retinal development in vivo has laid the foundation for using pluripotent stem cells to model retinal development in vitro.^42^ As a result, we can now generate human retinal neurons in almost unlimited quantities.^43–46^ Fundamental insights into human retinal development were gained by the discovery that in vitro pluripotent stem cells, when first differentiated into forebrain progenitors, will spontaneously self-organize into three-dimensional (3D) embryonic retinas. In the presence of basement membrane components, forebrain progenitors develop optic vesicle-like structures that invaginate to form bilayered optic cups that produce retinal neurons, including RGCs, in a histogenic sequence characteristic of the vertebrate retina in vivo.^42,47,48^ RGCs generated from these 3D retinas can grow long axons.^49,50^ When forebrain aggregates are grown in the absence of basement membrane components, they form 3D optic vesicle-like spheres that fail to undergo further morphogenesis, but nonetheless, differentiate into layered retinas, complete with RGCs and synaptic neuropil.^51–53^

Despite the striking recapitulation of retinal development in vitro, limitations of human 3D retinas are their slow development, which hews closely to the human developmental timeframe, and the genesis of retinal neurons in proportions that are present in vivo (where RGCs are less than 1% of the total). These features limit the utility of using retinal organoids to generate retinal neurons that may be used for transplantation. To overcome these limitations, approaches have been developed to differentiate human pluripotent stem cells directly into RGCs^54,55^ or convert cells isolated from the human retina into multipotent progenitor states and then into neurons, including RGCs. For example, human RPE contains a small population of self-renewing stem cells that can be differentiated into multiple cell lineages, including neuronal lineages.^56,57^ Müller glia isolated from the human retina can dedifferentiate in vitro into retinal stem cells, which can be propagated and redifferentiated into RGCs.^58,59^

### Regeneration of Retinal Neurons In Vivo

Unlike mammals, a small number of vertebrates have the capacity to spontaneously regenerate the retina. Two endogenous cellular sources can give rise to regenerated retina, the retinal pigmented epithelium (RPE) and Müller glia.^60^ The RPE originates from the same neuroectodermal lineage as the retina, but early in development the RPE adopts a nonneuronal function, which, from that point forward, supports aspects of retinal neurogenesis and, later, photoreceptor physiology. The ability of the RPE to regenerate retina is limited to some amphibians and the embryonic chick.^61–63^ In these models, after excision or degeneration of the retina, a subset of cells in the RPE detach from Bruch's membrane, expel their pigment granules and proliferate to create a retinal epithelium overlying the original, albeit repaired RPE. This epithelium then, following the embryonic histogenic pattern, differentiates into a fully functional retina, including a new optic nerve that innervates central targets. The signaling pathways that underlie the transdifferentiation of RPE into retinal progenitors are yet to be thoroughly described,^64,65^ though the initial signaling events are thought to be the loss of cell–cell and cell–basement membrane contacts, re-expression of transcription factors that specify a retinal identity and the activation of a fibroblast growth factor (FGF) signaling pathway.^63^ Interestingly, transdifferentiation of the RPE in the chick is transient and limited to early embryonic stages.

Müller glia are the only retinal glial cell that are derived from the retinal epithelium. Further, transcriptome analysis shows that mature Müller glia express numerous genes in common with mitotic, late-stage retinal progenitors.^66^ Therefore, it is perhaps not surprising that in some vertebrates Müller glia retain the ability to enter the cell cycle and, thereby, can function as an intrinsic retinal stem cell. The post-hatch chick and teleost fish have emerged as the most informative models for investigating the stem cell properties of Müller glia (reviewed in Refs. 67–71). In zebrafish, Müller glia sustain the persistent, growth-associated genesis of rod photoreceptors that is characteristic of teleost fish.^60^ In addition, Müller glia in zebrafish respond to the presence of dying neurons by partially dedifferentiating, undergoing a single asymmetric cell division,^72^ and spawning rapidly dividing progenitors that can regenerate a single subtype of retinal neuron following its ablation^73^ or regenerate an entire retina following extensive cell death.^74^ Presently, there is an intense research effort focused on deciphering the intercellular signaling pathways by which Müller glia become neurogenic when in the presence of dying cells. A key node in these signaling pathways is the proneural transcription factor Ascl1a. Within Müller glia, multiple pathways appear to converge on the regulation of this gene, and if the injury-induced upregulation of *ascl1A* is blocked, Müller glia fail to enter the cell cycle and neuronal regeneration is forestalled.^75^ In a recent study using medaka fish, a conditional gene expression paradigm was used to further investigate regenerative mechanisms in Müller glia. This study showed that in an uninjured retina, the conditional expression of *atoh7* was sufficient to force quiescent Müller glia into the cell cycle.^76^ Müller glia expressing *atoh7* gave rise to mitotic retinal progenitors that then differentiated into mature retinal neurons. Importantly, the supernumerary neurons generated by the forced expression of *atoh*7 were largely RGCs, a result that links *atoh7* both to the genesis and regeneration of RGCs.

In mammals, Müller glia are normally mitotically quiescent. In response to cell death, Müller glia become reactive and gliotic, but only rarely enter the cell cycle.^77^ Whether or not Müller glia have a native ability to effect neuronal regeneration is controversial.^70^ However, in mice, if neuronal death is coupled with the intraocular injection of growth factors, Müller glia will dedifferentiate, enter the cell cycle, and support modest neuronal regeneration.^78^

Further, if retinal cell death is combined with the conditional expression of *ascl1*, Müller glia initiate a regenerative response resembling that described for zebrafish.^79^ Importantly, in the absence of cell death, the forced expression of *ascl1* does not alter the Müller glia phenotype or induce entry into the cell cycle. Though this neuronal regeneration does not approach that observed in fish, it confirms that in mammals Müller glia in vivo can be induced to respond to cell death by adopting a neurogenic phenotype and regenerate retinal neurons.

Finally, recent studies show that in mammals, retinal neurons can be regenerated from an endogenous cellular source when cell death is coupled with cell fusion–mediated reprogramming. Cell–cell fusion, the merging of plasma membranes to integrate intercellular components, is a tightly regulated process that occurs naturally during development and in a variety of pathologies.^80^ Fusion in vivo between stem cells and adult somatic cells can reprogram somatic cells into multipotent progenitors.^81,82^ In a manner dependent on Wnt signaling, hematopoietic stem cells transplanted into lesioned retinas of adult mice will spontaneously fuse with host retinal neurons and Müller glia.^83,84^ When coupled with injuries that kill inner retinal neurons, the cell hybrids become mitotic, revert to a neuronal lineage, and differentiate into amacrine cells and RGCs.^83^ When coupled with the selective death of photoreceptors, hematopoietic stem cells fuse exclusively with Müller glia, which become neurogenic and selectively regenerate photoreceptors.^84^

While our knowledge of the mechanisms governing the development and regeneration of retinal neurons has advanced, many fundamental questions remain. Importantly, we still do not fully understand why there are profound differences in regenerative capacity across species, why this process is so limited in warm-blooded vertebrates, including humans, and how regeneration could be enhanced and optimized to effectively treat human disease. Therefore, the goal of this AGI workshop was to build upon our knowledge of retinal development and regeneration to delineate opportunities and barriers and to begin to map a path toward RGC replacement in human disease.

## Discussion: Gaps in Scientific Knowledge and Barriers to Progress

### Cell Sources for RGC Regeneration and Replacement

#### Exogenous Sources

While the focus of the workshop was on endogenous sources for RGC replacement, the group first discussed recent advances made in using exogenous sources to generate RGCs. Either human embryonic stem cells (ESCs) or induced pluripotent stem cells (iPSCs) were identified as a viable source for donor cells because they can be derived in unlimited numbers, and can be directed to generate retinal progenitor cells that under defined conditions differentiate into RGCs or other retinal cell types. Recent studies were discussed demonstrating that RGC-like cells derived from human iPSCs have morphological, phenotypic, and functional characteristics expected of RGCs (reviewed in Refs. 85–87). The panel identified some advantages to these approaches, including modeling aspects of human retinal development, as well as enabling the use of genetic tools and in vitro manipulations to promote RGC differentiation. For example, making use of reporter lines to identify cells that have undergone differentiation could help refine and optimize differentiation conditions.^55^

Ultimately, exogenous cells may also provide a source of storable tissue for transplant studies, although a number of challenges were highlighted. For example, it is still difficult to produce RGCs in numbers and over a time course that is scalable. In addition, the effect of freezing and storing cells prior to transplantation needs to be assessed. Moreover, because ESCs and iPSCs will differentiate into a heterogeneous variety of cell types, strategies need to be developed to either purify cells that will make RGCs, or more efficiently direct these cells to generate RGCs, for example by expressing specific transcription factors. Furthermore, it is currently unknown what stage of RGC development is best for transplantation, but photoreceptor replacement studies suggest that transplanted cells shouldn't be too primitive, but also shouldn't be too mature (reviewed in Ref. 88). It was also emphasized that the more general challenge of immune rejection will need to be addressed, unless cells are derived from that host, obtained from super donors with certain human leukocyte antigen (HLA) types, or engineered to evade immune detection.

One challenge to transplanting exogenous cells is to understand the role of the recipient environment, (e.g., damaged versus intact retina). It is still unknown whether signals in the recipient environment are sufficient to direct exogenous cells to differentiate as RGCs. For example, if RGCs are the primary cell type that is damaged or injured, will this help promote replacement of the cell that is normally there but now absent by disease, or preferentially generate cells in the area of the pathology? In zebrafish there seems to be propensity to replace missing cells, including some recognition of which cells are missing, with those being the ones regenerated in greater amounts (reviewed in Ref. 71). Whether the host environment will play a role in human transplant studies remains to be seen.

#### Endogenous Sources

The primary focus of the workshop was on utilizing endogenous sources for RGC replacement, building upon advances in retinal regeneration achieved in model systems, as highlighted above. There are multiple cell populations with the potential to contribute to retinal cell regeneration, including Müller glia, RPE cells, and stem cells within the ciliary epithelium. In considering which cell population to target for endogenous regeneration, the group emphasized the importance of understanding the mobility of cells and the cues that direct them to move through the retina. In particular, it will be important to define the cues that indicate loss of RGCs and trigger regeneration and control cell migration to the ganglion cell layer. Müller glia are among the most promising target for reprogramming due to their ability to contribute to retinal regeneration in species such as fish and post-hatch chick (see above). Furthermore, Müller glia–derived progenitors can move through the layers and potentially populate the RGC layer to become RGCs. Age may be a critical variable and may reflect epigenetic changes in Müller glia as the retina matures because, for mammals, Müller glia in younger retinas are more amenable to being redirected toward neural fates.^79,89^ In addition, much remains to be learned about how to more selectively direct Müller glia to regenerate RGCs.

While RPE cells in some contexts contribute to retinal regeneration, the potential for endogenous RPE cells to selectively replace RGCs was felt to be somewhat limited. In particular, it may be difficult to induce RPE cells in an endogenous intact retina to migrate through the retinal layers to the RGC layer. However, it was mentioned that in proliferative vitreoretinopathy, RPE cells can migrate through the retina and contribute to the formation of epiretinal membranes, which illustrates how RPE cells can respond and acquire different properties that could potentially be harnessed.^57,90,91^ It was noted that when transcription factors have been expressed in human RPE to push them into the neural lineage, they take on quite a different morphology and growth characteristics,^92^ suggesting they could be reprogrammed, although this has yet to be shown in an in vivo system selectively depleted of RGCs. The possibility of targeting ciliary epithelial stem cells was also addressed, although these cells would have to move from the margins of the eye to the ganglion cell layer. It is possible to direct these cells to differentiate into neurons. However, the cells retain pigment granules, so their application to RGC replacement may be limited.^93^

Other less conventional cell sources for RGC replacement were also discussed. For example, astrocytes can be redirected toward neuronal fates,^94,95^ and if in proximity to RGCs, they are theoretically targetable. One challenge is that astrocytes play important roles in the optic nerve, so redirecting them to alternate fates could be detrimental. However, it might be possible to use them as a source for replacing RGCs, while also decreasing potentially negative effects of depleting astrocytes. An innovative approach suggested targeting displaced amacrine cells, which already express Isl1 and Sox2, so they could potentially be redirected to an RGC fate. A recent study showed that Lgr5-positive amacrine cells have progenitor-like properties and generate new neurons in adult mice, so they may be amenable to RGC-directed reprogramming.^96^ This intriguing possibility was considered worth pursuing.

While retina regeneration studies have advanced in recent years, there is still a paucity of specific knowledge regarding the regeneration of RGCs, so the group discussed how the production of functional RGCs could be optimized. Overall, one clear priority is to thoroughly define the transcription factors and signaling pathways that promote efficient reprogramming of endogenous cells and RGC differentiation, particularly in humans. It was suggested that these efforts should consider the role of progenitor competence and timing, because during normal development RGCs are born during a restricted developmental window.^28^ One question that was raised is whether all RGC types need to be generated. If the goal is to help a nonseeing person to see, what characteristics of RGCs are necessary to process and transmit information? Current approaches address gene expression, morphology, and connectivity to mimic normal RGCs. But it is important to define what are the most important characteristics to mimic. It was suggested that it may be unnecessary to generate an exact RGC, but rather a neuron that has the essential functionality (i.e., a generic projection neuron). The essential functional characteristics are that it must project through the optic nerve, arrive at the correct targets, make appropriate afferent connections, and perform basic processing of visual information. It was recommended that fully characterizing human RGCs and defining requisite criteria should be a priority, including detailing functional properties and the array of molecular markers that are characteristic of RGCs.

### Optimizing Integration, Survival, and Synaptogenesis of New RGCs

#### Targeting and Delivery

The panel considered the best strategies for targeting endogenous cells and introducing genes or factors into the host retina for reprogramming. Gene therapy approaches in the eye have focused on viral vectors, particularly adeno-associated virus (AAV), for gene delivery.^97^ The group discussed efforts to optimize targeting of ocular tissues, and specific retinal types, by screening large libraries of AAV with novel changes in the capsid. Because there are species differences in the specificity and targeting of these vectors, these screens are being performed in rodents, canines, and primates. To optimize viral targeting of human cells, it was suggested that vectors could be screened on the human 3D eyecup in vitro. AAV variants have already been identified that specifically target Müller glia in the rodent retina. It may ultimately be possible to target each major subclass of RGC, as well as targeting other cell types such as RPE. One important consideration raised for reprogramming of cells is the timing of delivery of factors, because the timing and order of expression of new genes is likely to be critical for Müller glia reprogramming.^98^ It was pointed out that while much focus has been on AAV, because it has a simple genome, it is limited because it only packages 4.7 kilobases, so alternate vectors were discussed.^99^ Adenovirus was identified as better in that regard, but this virus puts major histocompatibility complex (MHC) on the outside of the cell, which can trigger a significant immune response, thereby limiting its application, particularly for individuals with systemic diseases.

#### Integration

For transplantation of cells from exogenous sources, an important consideration is how to enhance the delivery and integration of transplanted cells. It was suggested that it may help to temporarily make the inner limiting membrane more porous and better able to receive the cells delivered to the eye, which could increase the likelihood of functional integration of RGCs. The role of the host environment is also critical. If there is ongoing disease, will the transplanted cells die for the same reason as the original RGCs? Will it be possible to engineer cells that may be more resistant to the same lesion? It was pointed out that changes to the extracellular matrix, which is significant in degeneration, need to be considered when transplanting cells into the damaged retina. During the initial testing phase, it was suggested that investigators choose a disease model in which there is not ongoing injury or damage, or where the causal risk factor can be mitigated. A chronic disease like glaucoma might be risky to choose for proof-of-concept studies. In many cases, we do not fully understand the pathophysiology of several optic neuropathies, which poses challenges for restoring RGCs in a potentially hostile environment.

The panel pointed out that much remains to be learned about the long-term survival and stability of regenerated or transplanted RGCs, and that this may need to be optimized. To achieve this goal, it will be necessary to identify, visualize, and follow new cells over time. This may be challenging in humans, because it is not clear whether it will be safe to transplant cells expressing standard exogenous reporters (such as green fluorescent protein). It may be necessary to develop a technology to mark and visualize the cells in humans in a safe way, or introduce technology to eliminate the cells if something goes wrong. It was noted that even without visible reporters, advances in adaptive optics and other in vivo imaging technologies may enable visualization of new cells, but not necessarily whether they have made connections. For transplantation of cells from exogenous sources, the panel emphasized the importance of monitoring the integration of transplanted cells. What are the criteria to prove that the cell that is being imaged and believed to be integrated is really the cell that was transplanted? While fluorescent reporters are valuable, it is critical to control for cell fusion, and for transfer of material (e.g., protein or RNA) between transplanted cells and recipient host cells.^100,101^ These studies must also distinguish RGC replacement from rescue of remaining RGCs or other indirect effects. For example, transplanted cells may provide neurotrophic support and improve the function of remaining RGCs, which may be a desirable outcome, particularly if RGCs are partially damaged but still able to recover. However, it is important to understand mechanisms and optimize beneficial effects, perhaps by targeting the early stage of disease when there is only initial damage to RGCs.

#### Connectivity

The group next discussed another important consideration for successful regeneration or replacement of RGCs, optimizing synaptogenesis, and connectivity. The focus was on assays that reveal RGC features that are functionally relevant, such as synaptic integration into the inner plexiform layer (IPL), the electrophysiologic response to light, as well as axon growth and guidance to the correct targets in the brain. Because previous workshops tackled the complex challenges of regenerating the optic nerve and targeting the brain, this discussion focused on connectivity within the retina. To assess successful integration within the retina at a structural level, it was proposed that one could visualize and count the number of synapses and dendrites and assess the morphology of neurons formed. New fluorescent reporter approaches and imaging techniques should help in this visualization at nanoscale level. High-resolution in vivo imaging tools exist, but making them more available to all investigators would speed assessment and discovery. The group recommended that to make the best use of these imaging tools, common standards need to be developed to allow comparison of results. The panel noted that differences in RGC subtypes could be an important consideration, because survival, synaptogenesis, and connectivity could vary among subtypes. It may be important to consider whether regenerated RGCs recreate subtype-specific synaptic circuits within the IPL. Utilizing or generating reporters for each RGC subclass using CRISPR technology could investigate this. It was emphasized that if ex vivo screens are utilized to test and optimize parameters for integration, they need to be performed in a system where there is an IPL present to allow for appropriate connectivity. To optimize integration into human retina, high throughput screens could be performed using explants of human or monkey retina, or 3D stem cell–derived eyecups before testing in vivo in animal models. However, it will be first important to establish the best in vitro test to predict success in vivo.

### Approaches for Assessing the Outcomes of RGC Replacement Therapies

#### Assessing RGC Function

The final discussion focused on assessing whether successful regeneration or replacement of functional RGCs has been achieved. The panel emphasized the importance of standardized functional assays to monitor RGC responses and connectivity at the cellular level in vivo. Several strategies were discussed for probing new RGCs to determine whether they have integrated and connected. The goal is to visualize the cells that have been transplanted or regenerated, stimulate the cells, and know that they are the ones that are carrying the signal. In model systems, this could involve optogenetic approaches to noninvasively stimulate RGCs, and then perform electrophysiological recordings in the brain to assess whether stimulated cells have actually connected. It was pointed out that in the spinal cord transplant field, researchers are using chemical or optogenetic methods to reversibly silence transplanted cells to study their functional contributions, a strategy that could be applied to RGCs. In addition, it may be possible to determine whether new RGCs have formed dendritic connections in the IPL by stimulating photoreceptors and monitoring the responses of the new RGCs. To assess whether the cells are there and still alive, an innovative strategy could determine if mitochondria are flowing within RGC axons, because these organelles can be visualized. In general, the panel recommended the development of standardized criteria for successful integration to allow studies to be compared across labs.

#### Assessing Visual Function

The panel then grappled with the important question of determining whether treatments lead to improved visual outcomes. It is first important to decide if the goal is ambulatory vision or high-acuity vision. If ambulatory vision is the goal, then it was suggested that it might suffice to replace just a few subtypes of RGCs. One interesting proposal was to utilize intrinsically photosensitive RGCs to obviate the whole problem of connectivity on the afferent side. A related question is how many RGCs are needed to restore useful vision, and how should they be distributed across the retina. It was proposed that perhaps as few as 10,000 cells may be sufficient for useful vision, although it depends on the quality of vision that is desired. If the quality of vision that many of us enjoy is needed, then it will require many more cells. In sensory substitution experiments, a 256 × 256 grid of sensory input to the tongue is sufficient for a person to obtain enough information about the visual world to ambulate,^102^ so it may require fewer cells than usually considered necessary for ambulatory vision. It is also likely that the cortical fill-in phenomenon will permit scene recognition even in a limited visual field. It is known that patients with end-stage glaucoma can have a tiny amount of visual field and yet have useful vision.

The group also considered the challenge of detecting improvement of visual function after treatment. If the goal is to assess recovery of vision, it will be important to establish sensitive and quantitative functional criteria. Another consideration is selecting appropriate diseases, or stages of disease, to target for therapy in proof-of-concept studies. If relatively few RGCs are needed for light perception, than it may be difficult to detect improvement unless subjects (experimental animals or patients) start with no light perception and no evidence of RGC function.

The stage of disease has other potential implications for success of treatment, because retinal circuits undergo remodeling after extended periods of neuronal loss.^103^ This can impact target structures in the lateral geniculate nucleus and visual cortex. If the goal is to have new RGCs recreate stereotypical synaptic circuits, then either acute injury or early stage disease should be targeted for therapy to ensure that there has not been time for upstream and/or downstream remodeling of synaptic circuits. This will help ensure that the supporting structures are still present and there is no progressive damage. At the same time, the group recognized that studies should not focus on early disease because naturally occurring improvement could still take place, which could confound studies of efficacy. We summarize these challenges in Box 1.

A final point is that animal models do not always adequately represent humans, and that many aspects of disease are specific to humans. If, based on animal models, it appears that a therapy will be safe, then it will be necessary to empirically test it in a wide range of human diseases and at different stages of severity. In other words, clinical research must be included as part of the experimental pathway toward developing therapies.

The panel discussion ended by articulating key recommendations (Box 2) to accelerate progress toward functional RGC regeneration. There was strong consensus that while these are early days for RGC replacement and regeneration, key intermediate objectives could be achieved within a reasonable time frame. These intermediate objectives (Box 2) could set the course for a path forward, and would help accelerate discoveries to ultimately achieve RGC regeneration and restoration of vision in human disease.

**Table 1:**
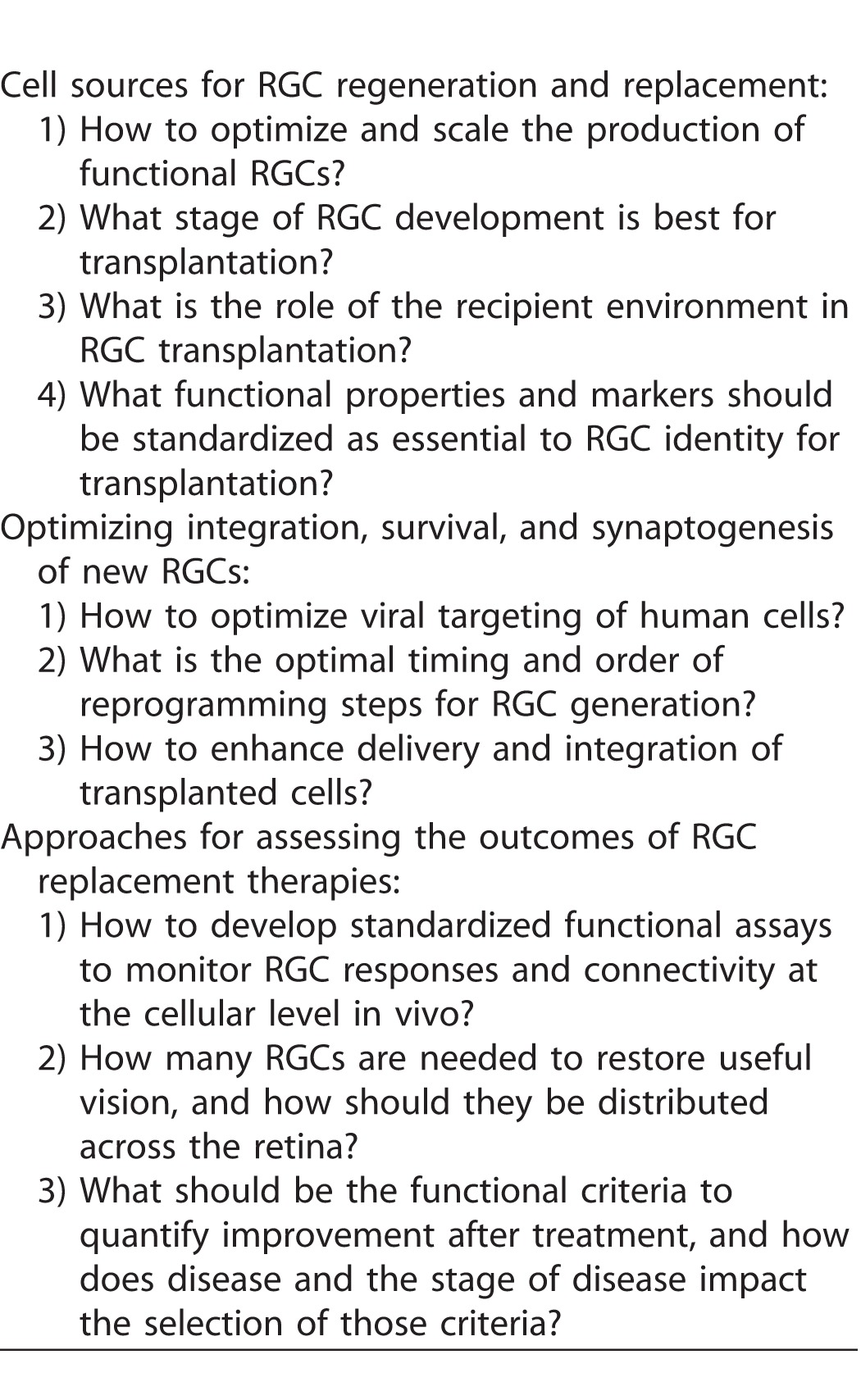
Summary of Gaps in Knowledge and Barriers to Progress

**Table 2:**
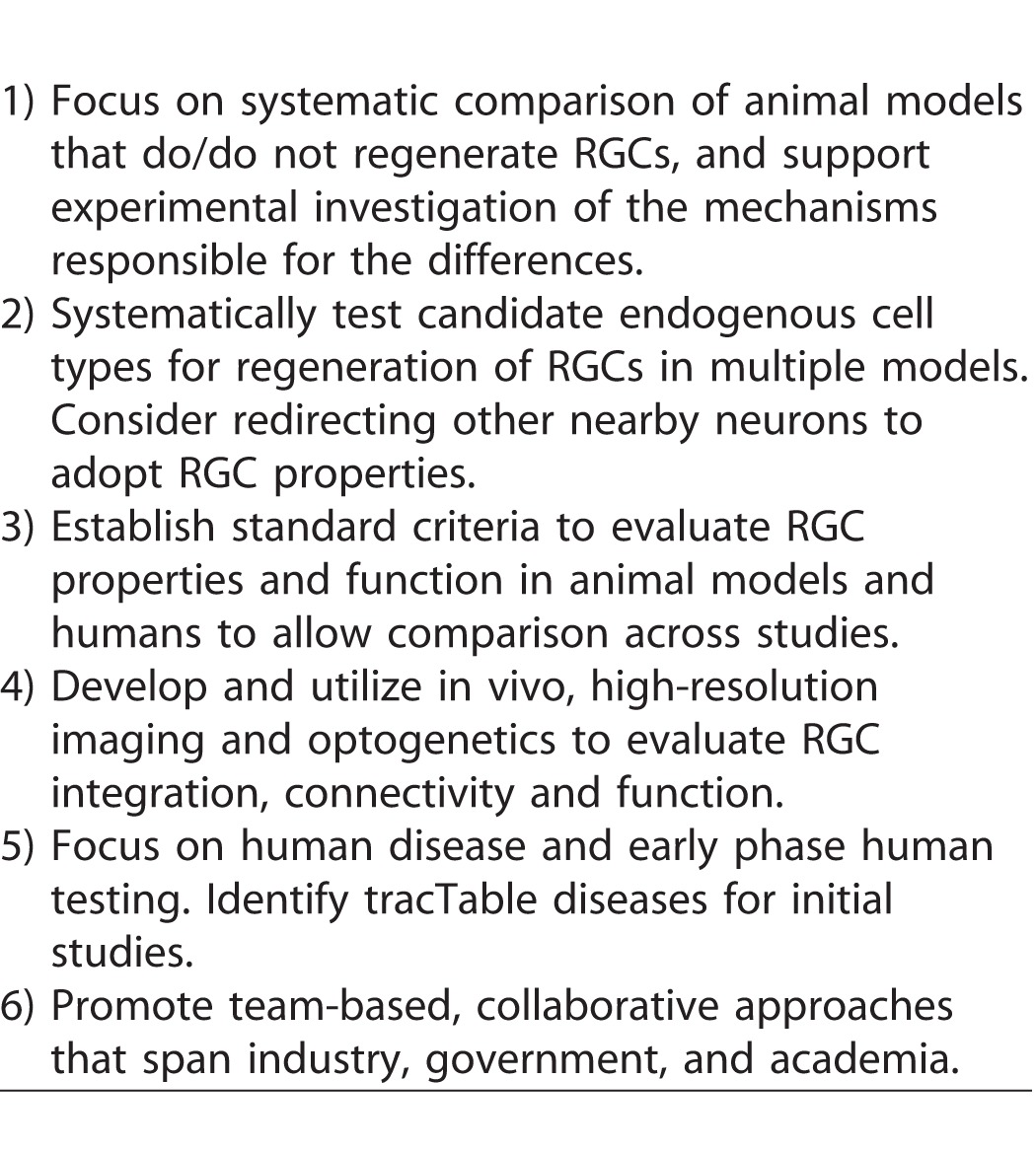
Key Recommendations to Accelerate Progress Toward Functional RGC Regeneration

**Table 3:**
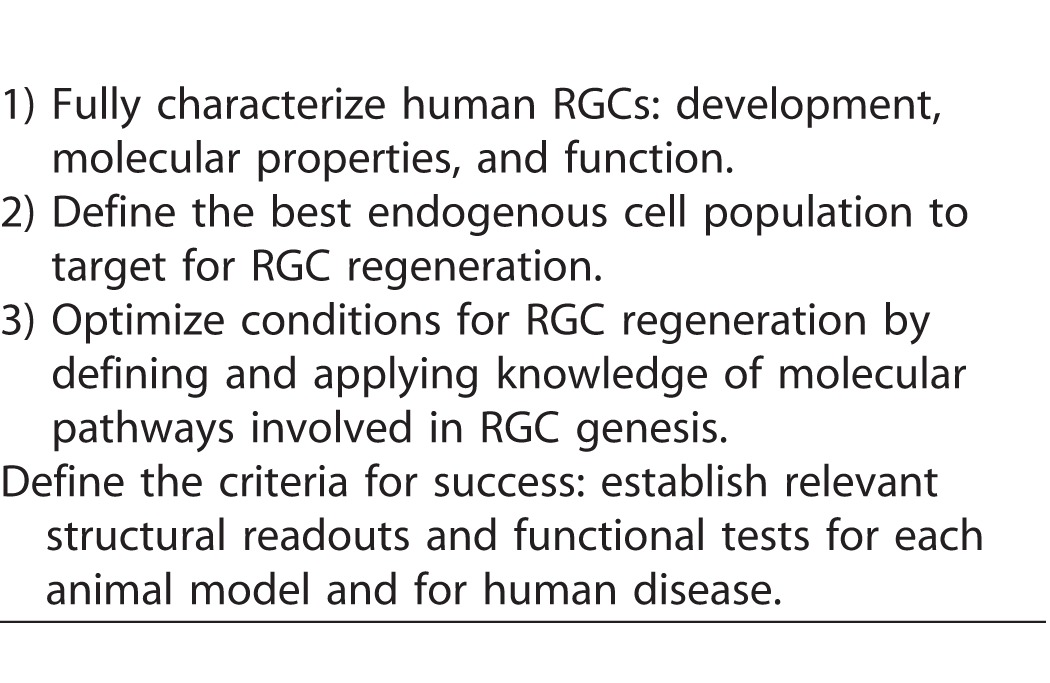
Intermediate Objectives

## Appendix A

Adah Almutairi, PhD

Associate Professor

Principal Investigator

Laboratory of Bioresponsive Materials

Co-Director

Center of Excellence in Nanomedicine & Engineering

University of California, San Diego

aalmutairi@ucsd.edu

Mark S. Blumenkranz, MD

Director

Byers Eye Institute

H.J. Smead Professor

Department of Ophthalmology

Stanford University

mark.blumenkranz@stanford.edu

Alain Chedotal, PhD

Director of Research

Institut de la Vision

Université Pierre et Marie Curie

alain.chedotal@inserm.fr

Maria Pia Cosma, ScD

ICREA Senior Investigator, Group Leader

Reprogramming and Regeneration Group Leader

Gene Regulation, Stem Cells and Cancer Program

Center for Genomic Regulation

pia.cosma@crg.eu

Andy Fischer, PhD

Professor

Department of Neuroscience

The Ohio State University College of Medicine

andrew.fischer@osumc.edu

John Flannnery, PhD

Professor

Vision Science, Neuroscience Division, Molecular and Cell Biology

Helen Wills Neuroscience Institute

University of California, Berkeley

flannery@berkeley.edu

Tom Glaser, PhD

Professor

Department of Cell Biology and Human Anatomy

School of Medicine

University of California, Davis

tmglaser@ucdavis.edu

Jeffrey Goldberg, MD, PhD

Professor and Chair

Department of Ophthalmology

Stanford University

jlgoldbe@stanford.edu

Peter Hitchcock, PhD *(Co-Chair)*

Professor and Associate Dean

Professor, Ophthalmology and Visual Sciences

Professor, Cell and Developmental Biology

Kellogg Eye Center

University of Michigan

peterh@umich.edu

David Hyde, PhD

Professor of Biology

Center for Stem Cells and Regenerative Research

University of Notre Dame

dhyde@nd.edu

Paul Kaufman, MD

Ernst H. Bárány Professor of Ocular Pharmacology

Departments of Ophthalmology and Visual Sciences and Animal Health and Biomedical Sciences

Institute on Aging

University of Wisconsin-Madison

paul.kaufman@wisc.edu

Leonard A. Levin, MD, PhD

Professor and Chair of Ophthalmology

McGill Academic Eye Centre

McGill University

Physician-in-Chief of Ophthalmology

McGill University Health Centre

leonard.levin@mcgill.ca

Astrid Limb, PhD

Professor of Retinal Stem Cell Biology and Therapeutics

Ocular Biology

Institute of Ophthalmology

Fellow

Royal College of Pathologists

g.limb@ucl.ac.uk

Jason Meyer, PhD

Assistant Professor

Stark Neurosciences Research Institute

University of Indiana – Purdue University, Indianapolis

meyerjas@iupui.edu

Xiuqian Mu, PhD

Associate Professor

Department of Ophthalmology

Jacobs School of Medicine and Biomedical Sciences

University at Buffalo, State University of New York

xmu@buffalo.edu

Yehoash Raphael, PhD

Professor

Department of Otolaryngology - Head and Neck Surgery

Kresge Hearing Research Institute

University of Michigan Medical School

yoash@umich.edu

Pamela Raymond, PhD

Stephen S. Easter Collegiate Professor

Department of Molecular, Cellular, and Developmental Biology

College of Literature, Science, and the Arts

University of Michigan

praymond@umich.edu

Douglas Rhee, MD

Professor and Chair

Department of Ophthalmology and Visual Sciences

University Hospitals Eye Institute

douglas.rhee@uhhospitals.org

Joel Schuman, MD

Professor and Chairman of Ophthalmology

New York University Langone Medical Center

joel.schuman@nyumc.org

Paul Sieving, MD, PhD

Director

National Eye Institute

National Institutes of Health

pas@nei.nih.gov

Sally Temple, PhD

Scientific Director

Neural Stem Cell Institute

Regenerative Research Foundation

sallytemple@neuralsci.org

Monica Vetter, PhD *(Co-Chair)*

Professor and Chair

Department of Neurobiology and Anatomy

University of Utah

monica.vetter@neuro.utah.edu

Valerie Wallace, PhD

Senior Scientist and Division Head

Krembil Research Institute

University Health Network

Department of Ophthalmology and Vision Science

Department of Laboratory Medicine and Pathobiology

University of Toronto

vwallace@uhnresearch.ca

Don Zack, MD, PhD

Co-Director

Johns Hopkins Center for Stem Cells and Ocular Regenerative Medicine

Professor of Ophthalmology

Johns Hopkins University

donzack@gmail.com
